# Incentives for orphan drug research and development in the United States

**DOI:** 10.1186/1750-1172-3-33

**Published:** 2008-12-16

**Authors:** Enrique Seoane-Vazquez, Rosa Rodriguez-Monguio, Sheryl L Szeinbach, Jay Visaria

**Affiliations:** 1 Division of Pharmacy Practice & Administration, College of Pharmacy, Ohio State University, Columbus, Ohio, USA; 2 Center for Health Outcomes, Policy & Evaluation Studies, College of Public Health, Ohio State University, Columbus, Ohio, USA; 3 School of Public Health and Health Sciences, University of Massachusetts, Amherst, Massachusetts, USA; 4 The Institute for Global Health, University of Massachusetts, Amherst, Massachusetts, USA

## Abstract

**Background:**

The Orphan Drug Act (1983) established several incentives to encourage the development of orphan drugs (ODs) to treat rare diseases and conditions. This study analyzed the characteristics of OD designations, approvals, sponsors, and evaluated the effective patent and market exclusivity life of orphan new molecular entities (NMEs) approved in the US between 1983 and 2007.

**Methods:**

Primary data sources were the FDA Orange Book, the FDA Office of Orphan Drugs Development, and the US Patent and Trademark Office. Data included all orphan designations and approvals listed by the FDA and all NMEs approved by the FDA during the study period.

**Results:**

The FDA listed 1,793 orphan designations and 322 approvals between 1983 and 2007. Cancer was the main group of diseases targeted for orphan approvals. Eighty-three companies concentrated 67.7% of the total orphan NMEs approvals. The average time from orphan designation to FDA approval was 4.0 ± 3.3 years (mean ± standard deviation). The average maximum effective patent and market exclusivity life was 11.7 ± 5.0 years for orphan NME. OD market exclusivity increased the average maximum effective patent and market exclusivity life of ODs by 0.8 years.

**Conclusion:**

Public programs, federal regulations, and policies support orphan drugs R&D. Grants, research design support, FDA fee waivers, tax incentives, and orphan drug market exclusivity are the main incentives for orphan drug R&D. Although the 7-year orphan drug market exclusivity provision had a positive yet relatively modest overall effect on effective patent and market exclusivity life, economic incentives and public support mechanisms provide a platform for continued orphan drug development for a highly specialized market.

## Background

On January 4, 1983, the Orphan Drug Act (ODA) went into effect to encourage the development and marketing of drugs (orphan drugs) to treat rare diseases and conditions. This Act evolved in response to the small number of orphan drugs that were approved in the U.S. in the years prior the approval of the ODA [1]. Between 1967 and 1983, an estimated 58 new drugs were approved in the U.S. that could have qualified for orphan status [2]

Initially, orphan drug status applied to products whose sales in the U.S. market would not cover the costs incurred during product development. However, opportunities to recover these costs improved when the 1984 ODA amendment expanded the definition of orphan drugs to include products for any disease or condition that affects less than 200,000 persons in the U.S. In January 2008, the National Institutes of Health (NIH) Office of Rare Diseases (ORD) listed 6,819 such rare diseases and conditions in the U.S., which afflicted an estimated 20 to 25 million Americans [3-5]. Approximately 250 new rare diseases and conditions are described each year [6].

In 1983, the ODA charged the FDA with the role of reviewing and approving requests for orphan product designation, overseeing the seven year exclusive marketing for orphan products, coordinating research study design assistance for sponsors of orphan drugs, encouraging sponsors to conduct open protocols, and awarding grants for development of orphan drugs. These functions are performed by the FDA Office of Orphan Products Development (OOPD). The Orphan Drugs Board in the Department of Health and Human Services was also established by the ODA to promote the development of orphan drugs and devices and to support a concerted effort between the public and private sectors in this area.

The ODA established federal status for orphan drug development, the so-called "orphan designation." OOPD's granting of orphan designation for a specific product indication qualifies the sponsor for incentives provided under the ODA. Although the original ODA allowed sponsors to apply for orphan drug designation at any time during product development or following FDA marketing approval, the Orphan Drug Amendment of 1988 allowed sponsors to apply for orphan drug designation at any time prior to the submission of a marketing application for the orphan indication. Thus, a sponsor may request orphan drug designation for any unapproved use of a drug without regard to whether other indications of the drug were approved previously for marketing.

Through the ODA, several economic incentives were created to stimulate orphan drug development and marketing. First, was the availability of grants, of which approximately 12 – 15 grants are awarded annually to academic-based researchers or to companies [8]. Second, was the establishment of a 50% tax credit for expenditures incurred during the clinical testing phase for orphan drugs being evaluated for their therapeutic potential. Congress made the tax credit permanent from May 31, 1997. The tax provisions also have a 20-year carry forward and a 1-year fall back provision. Third, was the 7-year market exclusivity provision granted for FDA-designated orphan drug indications [8]. The ODAs 7-year market exclusivity is a post-approval incentive that begins on the date of FDA approval for the designated orphan indication. This incentive addressed the limited opportunities to recover R&D costs for drugs without patent protection. However, exclusivity does not share the same level of protection as a patent [9]. During the orphan market exclusivity period, the FDA cannot approve a NDA (new drug application) or a generic drug application for the same product and for the same rare disease indication. The FDA could approve a second application for the same drug for a different disease indication.

Although previous studies evaluated the effects of the ODA on orphan drug designations and approvals, no studies have evaluated the patent and market exclusivity periods of orphan drugs [2,7,10-12]. Therefore, the objectives of this study were to analyze the characteristics of the orphan drugs designations and approvals, and their sponsors, and to evaluate the effective patent and market exclusivity life of orphan NMEs approved in the US market during the period 1983–2007.

## Methods

The primary data sources used in this study were: the FDA "Approved Drug Products with Therapeutic Equivalence Evaluations (i.e. Orange Book-OB) versions from 1983 to 2007, the electronic version of the OB, the FDA OOPD List of Orphan Designations and Approvals, documents and data from the FDA's website, and information about patents abstracted from the U.S. Patent and Trademark Office (USPTO) website. Data were updated through December 31, 2007. Data collection was based on a prospective protocol developed by the authors. To guarantee the reliability of the data collection, one researcher was responsible for primary data extraction and placement into evidence tables while a second researcher verified the data extraction and entry process. Discrepancies between the two researchers were discussed and resolved by a third researcher. Algorithms were also used to ensure the accuracy and consistency of the data extraction process.

Data used in the analysis covered all orphan designations and approvals/licenses listed by the FDA. The study also included all orphan and non-orphan NMEs approved by the FDA from 1983 to 2007. To be included in the analysis, a drug product had to be considered by the FDA as a NME, and listed in the first NDA approved by the FDA for the NME. Additionally, NMEs for drug products were excluded from the patent analysis if they were: 1) never marketed after FDA approval; 2) discontinued or withdrawn from the market; or 3) found not to have at least one patent listed in the OB at some point during the period of analysis.

The unit of analysis was the first NDA and the first NDA Product Number for each NME approved by the FDA during the study period. The FDA review time was estimated as the difference between the NDA approval date and the NDA received day. The effective patent and market exclusivity life includes the period from NDA approval to market exclusivity and patent expiration. Patents with the minimum and the maximum effective patent life were used to estimate the minimum (first patent) and maximum (last patent) effective patent and market exclusivity life when several patents were listed in the OB for a NME.

Summary descriptive statistics were computed for the variables included in the analysis. Differences in proportions were assessed using Chi-Square and Fisher's exact tests. Group differences were assessed using group comparison t-tests. Logarithm transformation was performed for non-normal distributed variables. SPSS version 16 was used for the analysis.

## Results

### Orphan designations and FDA approvals

On December 31, 2007, the FDA listed 1,793 orphan designations granted since 1983 (Table 1) [13]. These designations referred to 1,199 products and 889 diseases and conditions. Cancer was the main group of diseases targeted by orphan designations (31.7% of all designations). Orphan designations for HIV/AIDS represented 4.9% of total orphan designations during the study period.

**Table 1:** FDA Orphan Drug Designations, Approvals and NMEs. U.S. 1983–2007

**Year**	**Designations**	**Approvals**	**NMEs**
1983	1	2	1
1984	40	3	2
1985	48	7	3
1986	32	6	4
1987	59	9	4
1988	73	10	4
1989	76	12	3
1990	88	12	5
1991	79	13	7
1992	56	14	6
1993	63	13	6
1994	59	11	3
1995	55	11	5
1996	58	24	8
1997	52	19	4
1998	65	20	7
1999	73	19	6
2000	69	14	2
2001	75	6	3
2002	61	14	5
2003	95	14	4
2004	131	14	10
2005	123	20	6
2006	141	22	3
2007	121	13	4

Total	1793	322	115

The orphan designations were granted to 810 sponsors, which included 95 universities, hospitals, public organizations and individual sponsors. The majority of sponsors had a low number of orphan designations with 509 (62.8%) sponsors having only 1 orphan designation (Table 2). There was a single sponsor for 88.6% of the products and 77.9% of the diseases and conditions targeted by the orphan designations.

**Table 2:** FDA Orphan Drug Designations and Approvals. U.S. 1983–2007

**Number of Designations**	**Number of Sponsors**	**Orphan Designations**	**Orphan Designations per Sponsor**	**NDA Approvals**	**NDA Approvals per Sponsor**	**NDA Approvals per Designation**	**NME Approvals**	**NME Approvals per Sponsor**	**NME Approvals per Designation**
1	509	509	1.0	48	0.09	0.09	18	0.04	0.04
2	140	280	2.0	40	0.29	0.14	20	0.14	0.07
3	61	183	3.0	28	0.46	0.15	7	0.11	0.04
4	33	132	4.0	12	0.36	0.09	4	0.12	0.03
5	14	70	5.0	8	0.57	0.11	5	0.36	0.07
6	8	48	6.0	9	1.13	0.19	8	1.00	0.17
7	11	77	7.0	24	2.18	0.31	10	0.91	0.13
8	7	56	8.0	16	2.29	0.29	3	0.43	0.05
9	3	27	9.0	10	3.33	0.37	3	1.00	0.11
10 or more	24	411	17.1	127	5.29	0.31	37	1.54	0.09

Total	810	1,793	2.2	322	0.40	0.18	115	0.14	0.06

Between 1983 and 2007 the FDA approved 322 orphan drugs. The orphan FDA approvals included 72 biologicals (22.4%) and 250 drugs (77.6%). The approvals referred to 239 unique products including 52 (21.7%) biologicals and 188 (78.3%) drugs. The approvals targeted 238 different diseases and conditions. Cancer was the main group of diseases targeted by orphan approvals (25.5%). The approvals were concentrated among 155 (19.1%) sponsors that also had 43.2% of the total orphan designations.

The FDA approval rate varied with the number of orphan designations held by the sponsors. Sponsors with 1 orphan designation had a 9% FDA approval rate. Sponsors with more than 10 orphan designations had a 31% FDA approval rate. Overall, 8.3% of the sponsors that had 5 or more orphan designations accounted for 60.2% of the FDA approvals. After approval of the Orphan Drug Amendments of 1988, the average time from orphan designation to FDA approval was 4.0 ± 3.3 years (n = 290).

### New molecular entities orphan designations and approvals

The FDA approved 635 NMEs during the study period. The first NDAs for 115 (18.1%) NMEs were approved by the FDA for an orphan designated indication or indications (i.e. orphan NMEs). There were 8 orphan NMEs that had 2 orphan designations for the first NDAs approval. One of the orphan NMEs approved by the FDA in the study period did not have an associated orphan market exclusivity period listed in the OB.

Market approvals of orphan NMEs were concentrated in 83 companies that had 67.7% of the total orphan approvals and 29.0% of the total orphan designations. A higher percentage of orphan NMEs than non-orphan NMEs (i.e. other NMEs) was sponsored by companies that had only 1 NME approved during the study period (29.6% vs. 13.8%) (p < 0.001)

Statistically significant (p < 0.001) differences in favor of orphan NMEs were found for the use of any Fast Track or Subparts E/H review procedures (29.5% vs. 9.5%), and also for NMEs given priority review by the FDA (83.5% vs. 35.4%). After natural logarithm transformation to achieve normality of the data, significant differences (p < 0.001) in FDA review time were revealed between orphan NMEs (1.6 ± 1.4 years {mean ± standard deviation}) and other NMES (2.2 ± 1.7 years). However, difference in FDA review time between both groups of drugs was not statistically significant when the analysis was performed for subsets including priority review and standard review NMEs.

### NME patents and market exclusivity

The patent and market exclusivity analysis included 99 of the 115 orphan NMEs and 421 of the 520 other NMEs approved during the study period. Although the FDA approved 4 antibiotic orphan NMEs and 37 other antibiotic NMEs before the Food and Drug Administration Modernization Act of 1997 (FDAMA) during the study period, by regulation, antibiotics approved between 1983 and 1997 could not have patents or market exclusivity listed in the OB, other than orphan drug market exclusivity. Thus, antibiotics approved before FDAMA and products withdrawn from the market were excluded from analysis. Also, in the study period there were 12 orphan drug NME market discontinuations and 72 other NME market discontinuations (including 10 antibiotics). Safety was the reason for 16.7% (n = 2) of the orphan NMEs market discontinuations (aprotinin bovine and levomethadyl acetate hydrochloride) and the reason for 34.7% (n = 25) of other NMEs market discontinuations. A significantly (p < 0.001) lower percentage of orphan NMEs had patents compared to other NMEs listed in the OB (67.7% vs. 89.5%). The FDA granted pediatric market exclusivity to 9.1% of the orphan NMEs approved during the study period and to 24.7% of the other NMEs (p < 0.001). Market exclusivity (including orphan and Waxman-Hatch market exclusivities) represented the longest effective patent and market exclusivity period for a significantly (p < 0.001) higher percentage of orphan NMEs compared to other NMEs (37.4% vs. 15.4%).

The minimum effective patent and market exclusivity life (including orphan drug market exclusivity) was 9.9 ± 3.7-years for orphan NMEs and 10.5 ± 4.1 years for other NMEs (no statistically significant difference). The orphan drug market exclusivity provision increased the minimum effective patent and market exclusivity life of orphan NMEs by an average of 0.9 years.

The maximum effective patent and market exclusivity life (including orphan drug market exclusivity) was 11.7 ± 5.0 years for orphan NMEs and 13.9 ± 5.5 years for other NMEs (p < 0.001). The orphan drug market exclusivity provision increased the maximum effective patent and market exclusivity life of orphan NMEs by an average of 0.8 years.

There were 19 (19.2%) orphan NMEs and 171 (32.8%) other NMEs with generic competition in the study period. Orphan NMEs had significantly less (p < 0.01) generic competition than other NMEs. No generic or NDA competitors entered the market prior to the expiration of the Waxman-Hatch NME and the Orphan drug exclusivities. Generic competitors for orphan NMEs entered the market an average of 4.4 ± 3.9 years (range 0–11.5 years) after expiration of the orphan drug market exclusivity. However, there were 3 orphan drugs that had generic entry immediately after orphan drug market exclusivity expiration, and 3 other orphan drugs that had generic entry in less than one year after the expiration of orphan drug market exclusivity.

## Discussion

### Encouraging the development of orphan drugs

The study results revealed a modest impact of the 7-year orphan drug market exclusivity on the overall orphan NME drug patent and market exclusivity life. Nonetheless, there is general agreement that the ODA has encouraged sponsors to develop and market orphan drugs in the U.S. [2,7,11,14]. In the 25 years since the enactment of the ODA in 1983, there were 322 NDA approvals for orphan indications and diseases, representing a significant increase in the number of orphan drugs available in the market. Although orphan drug designations and approvals target a fraction of the almost 7,000 identified rare diseases and conditions, the approval of these products does not necessarily represent the real extent in which pharmaceutical R&D has benefited patients.

In spite of the positive effect of the ODA, it is not possible to estimate how many of the orphan drugs would have been approved in the absence of the ODA incentives [15]. For example, the OOPD had 15 sponsor commitments to submit marketing authorizations for orphan drugs before the approval of the ODA [16]. Other factors may explain the increase in the number of orphan drugs. First, public and private investment in pharmaceutical R&D increased during the same period [17]. This increase in R&D could facilitate the discovery and development of orphan drugs *per se *and as a byproduct of research for non-orphan diseases and conditions [18]. Second, other U.S. regulations, specifically the Waxman-Hatch Act and the Prescription Drug User Fee Act increased the general pharmaceutical patent and market exclusivity periods. Third, advances in science and technology, specially the development of the biotechnology industry also affected orphan drug R&D [7,9,17]. In fact, the first product brought to the market by top US biotechnology companies according to sales was an orphan drug [6]. Fourth, after approval of the Small Business Innovation Development Act of 1982, the NIH dedicated part of its R&D budgets to grants for small businesses, which are more prone to target orphan drugs. During the period 1982–2007, over $12 billion were awarded by the program to various small businesses in health care and other areas of research [19]. Fifth, several public initiatives including the Orphan drugs Board and the NIH ORD also encouraged development of orphan drugs. Sixth, orphan diseases patient groups advocating for orphan drug R&D have increased significantly during the study period [6]. Seventh, substantial changes in FDA review procedures occurred during the study period that reduced the time required for drug approval and allowed for limited commercial distribution of unapproved drugs [20]. Finally, the demand for orphan drug products may be increased through the effect of public programs that finance an important part of the inpatient and outpatient pharmaceutical expenditures in the U.S. [21].

### Understanding the sponsors

Companies representing smaller and newer entities, universities, and private investigators from independent entities have already been noted as OD sponsors [15]. This study found that almost two-thirds of the sponsors had only 1 orphan designation. The importance of the smaller sponsors is demonstrated by the fact that 75% of the diseases targeted by orphan designations had only one sponsor. This study also revealed the complex relationship between orphan sponsors and products, with products having several orphan designations, and with several sponsors and multiple orphan designations for the same sponsor [7,15].

Given that orphan drugs are often identified during studies to treat diseases in larger patient populations, greater participation by large companies with hundreds of products in development should be expected [9]. The relatively small number of orphan drugs developed by large companies may be explained by priorities that emphasize research toward drugs with a larger potential for profit [17,22]. The more efficient prioritization of R&D projects by large companies may also explain why large companies were more successful in achieving FDA approval for their orphan designations.

Also, the lower proportion of smaller companies with non-orphan NMEs could be attributed to the characteristics of these companies. In that small companies may be aligned to compete in broad therapeutic classes, but they are also able to target orphan drug niches, which may not require high R&D costs and large scale marketing efforts [23]. Furthermore, when an orphan drug is the only alternative available in the market for a disease, public and private programs are likely to facilitate the diffusion of information about the product to patients and health professionals, thus reducing marketing costs. If experience with the FDA approval process is a barrier, the smaller companies may benefit from the FDA research design assistance program for orphan drug development [9]. Clearly, future research is needed to better understand the relationship among drug sponsors, products and the incentives for orphan drug R&D options with respect to the dynamics of their existing environment.

### The role of economic incentives

The FDA did not establish special procedures or safety and efficacy standards for approval of orphan drugs; nonetheless, orphan drugs had shorter development time than other drugs. The FDA has been flexible in the requirements for approval of orphan drugs, especially for drugs designated to treat serious or lifethreatening illnesses [20,24]. Under these circumstances, since the majority of orphan products were approved to treat serious and life threatening illness or to provide a treatment where no adequate therapy exists they were more likely to be approved using the accelerated, Fast Track and priority review procedures [10]. Moreover, resources allocated by the public sector to support orphan drug R&D allow single-product sponsors to participate in orphan R&D that, otherwise, could not assume the risk and opportunity cost associated with developing such products [7]. While different sources report varying levels of orphan drug approvals in oncology and other therapeutic areas [25], for this study approximately 18% of the orphan drug designations between 1983 and 2000 were approved by the FDA.

Tax incentives also reduce R&D costs, especially for established for profit sponsors. The tax incentives operate when the company that is developing the orphan drug has income from sales of other products and from commercial distribution of unapproved orphan drugs or royalties. Hence, tax credits and FDA fee waivers may serve as incentives for sponsors to apply for orphan designation in cases where the seven-year orphan drug market exclusivity period is superseded by the effective patent life. Grants also facilitate the participation of sponsors that develop orphan drugs but do not market them. Although grants permit the prioritization of R&D for orphan drugs with input from the public sector, these grants are primarily given to academic-based researchers and less frequently to pharmaceutical companies.

From the consumers' perspective, taxpayers could end up paying twice for orphan drugs. First, taxpayers may end up paying for orphan R&D through grants, tax credits, and the cost of FDA and other public agencies support to sponsors. Second, later payments for the cost of orphan drugs appear in public health care programs such as Medicare and Medicaid. Despite the economic and marketing tradeoffs, the ODA has ensured the development and marketing of orphan drugs in an environment driven by consumer demand for treatments where no adequate therapy exists.

### Orphan drug market exclusivity

This study estimates and compares the barriers to competition added by the Orphan Drug Act Exclusivity, the Waxman-Hatch Act exclusivity and the patent life. These three barriers are technically different. The patent life offers the broadest level of protection and patents may cover pharmaceutical compositions, indications or uses, dosage forms, or manufacturing processes. A valid patent protects a drug product from both generic and full NDA FDA approvals. Nevertheless, patents listed in the Orange Book are often invalid or do not protect the drug from generic or full NDA competition [12]. The Orphan Drug Exclusivity offers the second broadest level of protection because the provision protects the Orphan designated indication against generic and full NDA approval. After the introduction of 5-year market exclusivity under the Waxman-Hatch Act in 1984, the additional exclusivity granted to NMEs by the orphan drug exclusivity is only two years. Yet the Waxman-Hatch Act is the less restrictive toward the barriers to competition because the Act protects NDAs only against generic competition. In practice, however, these exclusivities are the most restrictive toward the barriers to competition as demonstrated by the fact that no NME generic or full NDA competitor was approved before the expiration of those exclusivities. The FDA monitors the status of the Waxman-Hatch Act exclusivities and the patents listed in the OB before considering a generic application for approval. The FDA also monitors the status of the Orphan drug exclusivity before considering a generic or full NDA application for approval.

The relatively low percentage of NMEs with generic entry immediately after the expiration of orphan drug exclusivity indicates that patents and other market factors, such as small patient populations and low expected profits, are also important barriers to generic entry. In fact, taking into account that only one in seven drugs had more OD exclusivity than patent life, and that only 1 in seven of those drugs OD exclusivity been the largest protection had generic competition; only 1 in 10 NME Orphan drugs benefited directly from the ODA exclusivity. These results contrasted with previous findings that indicated that the orphan drug market exclusivity provision was the strongest of ODA incentives [15,26].

## Conclusion

Public programs, federal regulations, and policies support orphan drugs R&D. Grants, research design support, FDA fee waivers, tax incentives, orphan drug market exclusivity, and public diffusion of orphan innovation are main incentives for orphan R&D. Most of these incentives were established by the ODA in 1983. Other factors including the expanding role of the NIH, scientific advances and other drug and patent regulatory changes also could explain the increase in orphan approvals that occurred in the past 25 years.

A large number of small sponsors also participate in orphan drug R&D and marketing. Grants and other public support programs are efficient ways to reduce the financial risk associated with orphan drug R&D. At the same time, the competitive nature of the US grant system allows for public prioritization of orphan drug R&D. As revealed in this study, orphan NMEs had significantly shorter FDA review time because a higher percentage of orphan drugs were approved under priority review and accelerated review procedures.

Despite these advantages, orphan NMEs had a statistical significant less effective patent and market exclusivity life than other NMEs. However, orphan NMEs also experienced less generic competition than other NMEs. In summary, the 7-year orphan drug market exclusivity provision had a positive yet relatively modest overall impact on effective patent and market exclusivity life. Besides consumer demand for innovative new drug products and devices, supply-side efforts such as grants, FDA fee waivers and tax credits may explain why drugs with orphan designation is pursued in the marketplace.

## Abbreviations

ODs: orphan drugs; NMEs: new molecular entities; FDA: Food and Drug Administration; R&D: research and development; ODA: Orphan Drug Act; NIH: National Institutes of Health; ORD: Office of Rare Diseases; OOPD: Office of Orphan Products Development; USPTO: U.S. Patent and Trademark Office; FDAMA: Food and Drug Administration Modernization Act; NDA: new drug application.

## Competing interests

The authors declare that they have no competing interests.

## Authors' contributions

ESV conceptualized the study, designed the study, performed the statistical analysis, and drafted the manuscript. RRM collaborated in the conceptualization and design of the study, performed the statistical analysis, and drafted the manuscript. SLS collaborated in the study design and the statistical analysis, and drafted parts of the manuscript. JV collaborated in the statistical analysis and in drafting parts of the manuscript. All authors approved the manuscript.
